# Health utilities for non‐melanoma skin cancers and pre‐cancerous lesions: A systematic review

**DOI:** 10.1002/ski2.51

**Published:** 2021-06-04

**Authors:** C. So, A. E. Cust, L. G. Gordon, R. L. Morton, K. Canfell, P. Ngo, M. Dieng, K. McLoughlin, C. Watts

**Affiliations:** ^1^ Sydney School of Public Health Faculty of Medicine and Health, The University of Sydney Sydney Australia; ^2^ The Daffodil Centre The University of Sydney, a joint venture with Cancer Council NSW Sydney Australia; ^3^ Melanoma Institute Australia The University of Sydney Sydney Australia; ^4^ Population Health Department QIMR Berghofer Medical Research Institute, Royal Brisbane Hospital Brisbane Australia; ^5^ School of Nursing Queensland University of Technology (QUT) Brisbane Australia; ^6^ School of Medicine The University of Queensland Brisbane Australia; ^7^ Faculty of Medicine and Health NHMRC Clinical Trials Centre, The University of Sydney Sydney Australia; ^8^ Kirby Institute The University of New South Wales Sydney Australia

## Abstract

**Background:**

Non‐melanoma skin cancers (NMSCs) are common and consume many healthcare resources. A health utility is a single preference‐based value for assessing health‐related quality of life, which can be used in economic evaluations. There are scarce data on health utilities for NMSCs.

**Objectives:**

Using a systematic review approach, we synthesized the current data on NMSC‐related health utilities.

**Methods:**

A systematic review of studies of NMSC‐related health utilities was conducted in Medline, Embase, and Cochrane databases. Data were extracted based on the protocol and a quality assessment was performed for each study.

**Results:**

The protocol resulted in 16 studies, involving 121 621 participants. Mean utility values across the studies ranged from 0.56 to 1 for undifferentiated NMSC, 0.84 to 1 for actinic keratosis, 0.45 to 1 for squamous cell carcinoma, and 0.67 to 1 for basal cell carcinoma. There was considerable variability in utilities by type of cancer, stage of diagnosis, time to treatment, treatment modality, and quality of life instrument or method. Utility values were predominantly based on the EuroQol 5‐dimension instrument and ranged from 0.45 to 0.96, while other measurement methods produced values ranging from 0.67 to 1. Lower utility values were observed for advanced cancers and for the time period during and immediately after treatment, after which values gradually returned to pre‐treatment levels.

**Conclusions:**

Most utility values clustered around relatively high values of 0.8 to 1, suggesting small decrements in quality of life associated with most NMSCs and their precursors. Variability in utilities indicates that careful characterization is required for measures to be used in economic evaluations.

1

**What is already known about this topic?**

- Non‐melanoma skin cancers (NMSCs) are common and consume many healthcare resources. Health utilities are often used in economic evaluations of healthcare programs, however, there are scarce data on health utilities for NMSCs.

**What does this study add?**

- The systematic review suggests that most utility values clustered around relatively high values of 0.8 to 1, suggesting small decrements in quality of life associated with most NMSCs and their precursors. There was substantial variability in the utilities depending on cancer subtypes, treatments and methods, therefore, developing a comprehensive catalogue of NMSC health utilities is crucial for future health economic evaluations to inform policy and resource allocation decisions related to skin cancer early detection and management.

## 2 INTRODUCTION

Non‐melanoma skin cancers (NMSCs), comprizing mostly basal cell carcinomas (BCCs) and squamous cell carcinomas (SCCs), are the most common cancers in countries with fair‐skin populations.^1^ In Australia, clinical management of BCCs and SCCs accounted for 8.1% of Australian health system spending on cancer in 2008–2009 (excluding screening).^2^ The incidence of BCCs and SCCs is increasing in many countries, and the caseload is further impacted by ageing populations.^3^, ^4^, ^5^, ^6^ NMSCs are often under‐reported as many cancer registries do not routinely gather this information^7^, ^8^ or the data collected is not separated by NMSC subtypes.^9^

While most NMSCs (except Merkel cell carcinoma) are associated with a lower mortality rate than melanoma,^2^ mortality rates for SCC are higher when diagnosed at a later stage,^10^, ^11^, ^12^ therefore, in principle, early detection of skin cancer is beneficial. A higher recurrence rate of BCC and SCC and higher metastatic rate for SCC is generally expected for larger tumour size at diagnosis or those with high‐risk features.^10^, ^13^ While uncomplicated and small tumours may be treated by cryotherapy, cautery, curettage or excision, more advanced cancers may require specialized procedures such as advanced reconstructive surgery, radiotherapy, chemotherapy and immunotherapy.^2^, ^14^ The potential benefits of a proposed screening or treatment should be balanced against the healthcare harms and economic costs, including those arizing from biopsies and surgical procedures, to determine the best use of limited healthcare resources. This is particularly pertinent for most NMSCs for which the mortality rates are relatively low, as benefits are unlikely to be reflected in reduced mortality but rather in improved quality of life.

Ascertaining health‐related quality of life (HRQoL) is important to evaluate patient perspectives of benefits and harms of detection and treatment, which may include scarring, pain, post‐operative complications and worry.^15^ The domains of HRQoL are usually measured by generic instruments such as the EuroQol 5‐dimension instrument^16^ (EQ‐5D), or dermatology‐specific instruments, such as Skindex,^17^ the Basal and Squamous Cell Carcinoma QoL (BaSQoL) questionnaire,^18^ and the Dermatology Life Quality Index (DLQI).^19^ As a component of assessing HRQoL, it is often useful to generate a single preference‐based value, called a health utility, combining all aspects of a health state that is measured on a scale from 0 (corresponding to death) to 1 (best possible health). The methods for obtaining health utilities are usually either direct, such as the time trade‐off and standard gamble methods,^20^ in which study participants indicate their preferences from a range of health states or scenarios,^21^ or indirect, using HRQoL instruments, such as the EQ‐5D.^16^ Additionally, some non‐preference based domains of HRQoL can be mapped to health utility scores using a validated algorithm.^22^, ^23^

Economic evaluations of healthcare programs commonly use health utilities to obtain quality‐adjusted life years (QALYs), a measure that adjusts survival time by the quality of life within a health state.^24^, ^25^ An advantage of this approach is the ability to objectively assess differences in costs and QALYs across healthcare interventions, allowing policy‐makers to compare the cost‐effectiveness of healthcare programs to inform resource allocations, across different diseases.^24^

Currently, there are limited data on health utilities for NMSCs.^26^ This systematic review was undertaken to provide synthesized information for future health economic evaluations and to highlight the gaps in the current evidence.

## 3 MATERIALS AND METHODS

### 3.1 Search strategy

A search strategy was developed and encompassed literature on NMSCs and HRQoL. NMSCs were searched using general keywords and keratinocyte cancer as well as specific keywords for SCC, BCC, actinic keratosis (AK) and solar keratosis (Table S1, Appendix S1). Studies that included rarer NMSCs (such as Merkel cell carcinoma) were included if identified in the search but we did not include specific keywords for them. Keywords and MeSH terms were incorporated into the search (Table S1, Appendix S1). MEDLINE, Embase, and Cochrane Database of Systematic Reviews were searched from inception to April 2020. Results were limited to English language. The reference lists of relevant articles were hand‐searched. The grey literature was not searched.

### 3.2 Study selection

The inclusion criteria were full‐text research articles: (1) referring to patients with cutaneous NMSCs, specifically BCC, SCC (including Bowen’s disease or intraepithelial carcinoma) and their precursors (e.g., AK), and other rarer NMSCs such as Merkel cell carcinoma, and (2) evaluating health utilities of patients using direct or indirect methods. Although AK is not considered malignant, it was included in the search due to being a clinically detectable precursor lesion of SCC.^27^ Authors of relevant conference abstracts were contacted to obtain full text or data.

Titles and abstracts were initially screened (by CS) and full text articles of the short‐listed studies were examined (by CS) with reference to the inclusion criteria.

Data extraction was independently performed (by CS and CW). Data extracted included: study population and setting, number of participants, mean age and gender, the instrument or method used to measure quality of life, utilities and any additional measures reported. Where treatment was specified, the subgroup numbers, treatment administered and the temporal relationship between treatment delivery and utility measurement were recorded.

Quality analysis of the studies was based on published guidelines for the conduct of systematic reviews of health state utility values.^28^, ^29^ The ROBINS‐I risk of bias tool^30^ was used to assess for bias due to missing data, as this is a common issue in quality of life studies. Where primary outcome data were not available, the authors were contacted for clarification.

The systematic review protocol was registered on the Prospero database (CRD42020179776). The PRISMA statement^31^ was followed for conducting and reporting the study.

## 4 RESULTS

### 4.1 Study characteristics

The initial search identified 589 studies, of which 116 were included from abstracts; 93 underwent full‐text assessment (Figure S1). Five additional studies were identified through the reference lists of relevant studies. The main reason for exclusion (120 studies) was the use of instruments from which utilities could not be derived, with the Skin Cancer Index, DLQI, and Skindex being the most common of such instruments.

Sixteen studies involving 121 621 participants met the inclusion criteria (Table 1). Of these, nine studies^32^, ^33^, ^34^, ^35^, ^36^, ^37^, ^38^, ^39^, ^40^ used EQ‐5D, four^41^, ^42^, ^43^, ^44^ used the time trade‐off method, and three^45^, ^46^, ^47^ used standard gamble.

**TABLE 1:** Summary of included studies

Authors	Year	Country	Descriptive Summary	Setting	Number of Participants (n)	Mean Age	% Male	Summary of Health States Studied	Instrument Used	Notes
Bertino et al^32^	2016	Italy, Slovenia, Spain, The Netherlands, Denmark, and United Kingdom	Patients with head and neck cancers at six European institutions who were deemed not suitable for standard treatment (surgery or radiotherapy) due to risk of functional loss or comorbidities. Tumour types: 32% BCC, 48% SCC, 9% melanoma, and 11% other. Treated with electrochemotherapy and bleomycin.	Hospital clinic	105	77	71	NMSC, pre‐ and post‐treatment	EQ 5D	Patients reviewed 1–2 months after treatment. If recurrence, then 2 months follow‐up and additional treatment. If no recurrence, then follow‐up at 4, 8 and 12 months. HRQoL administered at each time point.
Chen et al^41^	2004	United States	Patients with dermatological conditions at two hospitals and a medical centre	Hospital, medical centre	250	46	43	NMSC, AK, pruritis, pre‐treatment	TTO	Utility values obtained prior to clinic appointment where diagnosis or treatment plan may not have yet been established.
Hanke et al^33^	2016	United States	Participants with AK in a randomized control trial. 167 patients with 4–8 AKs requiring treatment. Phase III, randomised‐controlled trial using cryotherapy and ingenol mebutate.	Clinical trial in the community	329	67	82	AK, pre‐ and post‐treatment	EQ 5D 3L	At least 89% of participants have had previous treatment at baseline.
Ker et al^34^	2019	New Zealand	Patients treated by split‐skin grafting for lower limb skin cancers from a single‐centre trial. 76% of participants had NMSC. Tumour types: 29% SCC, 43% BCC, 16% melanoma, 4% keratoacanthoma, 6% dermal scar, 2% other.	Hospital	49	71	53	NMSC, post‐treatment	EQ 5D 3L	Utility values were obtained at day 5–7 when dressing change was initiated prior to discharge. Patients followed for 3 months.
Lear et al^45^	2008	Canada	Referred patients to tertiary care, hospital‐based, skin cancer clinic.	Hospital	41	60–69^a^	44	BCC, SCC; pre‐ and post‐treatment	SG^c^	Time period after treatment not explicit.
Littenberg et al^46^	2003	United States	General dermatology patients attending dermatology outpatient clinics.	Dermatology clinic	74	52	24	NMSC, AK	SG^d^	Clinically stable outpatients enrolled before their scheduled dermatology clinic review. Uncertain if NMSC/AK had been treated at time of participation.
Philipp‐Dormston et al^35^	2018	Germany	Patients with NMSC from local medical practices and hospitals in Germany. Tumour types: 73% AK, 49% BCC, 17% SCC. Current treatment: 56% physical treatment, 21% drug therapy, 29% photodynamic therapy, 9% ‘watch and wait', 10% no treatment.	Hospital, local medical practice	1184	74^a^	61	AK, SCC, BCC	EQ 5D 5L	Patients followed for duration of treatment (median: 49 months). Questionnaire completed on enrolment at time of diagnosis. Patients at various stages of treatment were recruited. QOL values adjusted for age
Pil et al^36^	2016	Belgium	Patients with NMSC from hospitals and private practices in Belgium. Treatment unspecified.	Hospital, private practice	287	61–70^a^	44	SCC, pre‐ and post‐treatment	EQ 5D	Based on patient questionnaires for some subgroups. Belgian population baseline utility of 0.81 applied to some subgroups.
Seidler, Bayoumi et al^42^	2012	United States	Dermatology patients interviewed consecutively from two hospitals and a medical centre	Hospital, medical centre	283	45^a^	44	AK, BCC, pre‐treatment	TTO	Data was from the same study population as Chen et al.
Seidler, Bramlette et al^43^	2009	United States	Consecutive sample of patients with NMSC on face and ears undergoing Mohs surgery at a tertiary care referral centre. Tumour types: 79% BCC, 21% SCC.	Hospital clinic	98	68	57	NMSC, post‐treatment	TTO	Time after treatment not explicit in the study, however implied shortly after treatment as no mention of loss to follow‐up rate.
Shingler et al^44^	2013	United Kingdom	Sample of the general public	General population	100	39	43	BCC, post‐treatment	TTO^b^	
Sullivan, Ghushchyan^37^	2006	United States	Based on the Medical Expenditure Panel Survey, a national survey of the general population	General population	38678	40–49^a^	48	Malignant neoplasms	EQ 5D 3L	Only included chronic conditions (>1 year duration)
Sullivan, Slejko et al^38^	2011	United Kingdom	Based on the Medical Expenditure Panel Survey, a national survey of the general population	General population	79522	40–49^a^	48	Malignant neoplasms, non‐epithelial skin cancer	EQ 5D 3L	Only included chronic conditions (>1 year duration)
Tennval et al^39^	2015	Denmark	Patients with AK in dermatological clinics. 55% had comorbidities.	Dermatology clinic	312	71	49	AK, pre‐ and post‐treatment	EQ 5D 5L	EQ 5D 5L completed on enrolment. Various stages of AK and treatment. No follow‐up.
Wali et al^40^	2020	United Kingdom	Patients with NMSC referred to dermatology clinics. 45% had moderate/severe comorbidities; 28% had mild comorbidities.	Dermatology clinic	279	74	61	NMSC, post‐treatment	EQ 5D 5L	Most utility values obtained after treatment
Wong et al^47^	2014	New Zealand	Convenience sample of patients with stage N0 cutaneous SCC of the head and neck who have already been treated with local excision	Hospital clinic	30	61^a^	73	SCC, post‐treatment	SG^d^	Time period after treatment not explicit.

Abbreviations: NMSC, non‐melanoma skin cancer; BCC, basal cell carcinoma; SCC, squamous cell carcinoma; AK, actinic keratosis; TTO, time trade‐off; SG, standard gamble; EQ‐5D (3L or 5L), EuroQol 5‐dimension instrument (3‐level or 5‐level).

^a^ Median value.

^b^ Using hypothetical health states.

^c^ Between two hypothetical scenarios: BCC on the nose and SCC on the lip.

^d^ Between cure of patient's current condition vs death.

BCC, SCC and AK were the most common conditions with some studies reporting pooled utility values from different types of NMSC without distinguishing the case‐mix. Only one study^36^ specifically referred to Bowen’s disease (SCC in situ), and no studies considered Merkel cell carcinoma.

Studies were based on populations from the United States,^33^, ^37^, ^41^, ^42^, ^43^, ^44^, ^46^ Europe,^32^, ^35^, ^36^, ^39^ the United Kingdom,^32^, ^38^, ^40^ New Zealand^34^, ^47^ and Canada.^45^ The sample size for studies varied from 30 to 1184 participants and population‐based datasets ranged from 38 678 to 79 522 subjects. Most studies, except one,^40^ performed subgroup analyses on type or severity of cancer, treatment, or time after treatment. Nine studies reported the mean age of participants,^32^, ^33^, ^34^, ^39^, ^40^, ^41^, ^43^, ^44^, ^46^ which ranged from 39 to 77 years; seven studies reported the median age,^35^, ^36^, ^37^, ^38^, ^42^, ^45^, ^47^ which ranged from 40 to 74 years. The gender distribution ranged from 24% to 82% male. Two studies^39^, ^40^ reported the proportion of comorbidities among participants, although the specific comorbidities were not specified.

Five studies^33^, ^39^, ^40^, ^44^, ^46^ were conducted in, or used treatments suitable for use in the community, and five studies^32^, ^34^, ^41^, ^45^, ^47^ were in hospital settings. Four studies^35^, ^36^, ^42^, ^43^ drew participants from both the community and hospitals. Two studies were national population‐based studies.^37^, ^38^ One study was a phase III clinical trial.^33^

Out of the 15 studies that reported mean utilities, six^32^, ^33^, ^35^, ^37^, ^38^, ^41^ also reported median values (which were greater than the mean in all subgroups of four of the studies^32^, ^33^, ^35^, ^41^), one^36^ also used assigned or calculated utilities, and seven^33^, ^34^, ^40^, ^41^, ^44^, ^45^, ^47^ also reported standard deviations. One study^42^ only reported median utility values.

### 4.2 Quality of studies and risk of bias

Six studies had a population sample size of 100 or less. For risk of bias due to missing data,^30^ 11 studies were deemed low risk, two were moderate risk, two were serious risk, and one had insufficient information for assessment (Table S2). Studies that were labelled as moderate or serious risk for missing data had low response or participation rates.

### 4.3 Categorization by skin cancer group and treatment

Studies that reported pooled utility values without distinguishing the types of skin cancer were categorized as NMSC, otherwise they were grouped by type of skin cancer.

#### 4.3.1 Undifferentiated non‐melanoma skin cancer

Nine studies obtained health utilities for NMSC (Table 2).^32^, ^34^, ^35^, ^37^, ^38^, ^40^, ^41^, ^43^, ^46^ The mean utility ranges were 0.72 to 1 and 0.56 to 1 for untreated NMSC (or where treatment was unspecified) and treated NMSC, respectively. Two studies^32^, ^34^ included patients with melanoma in their analysis, however the proportion of melanomas was relatively low (range 9%–16%). The NMSC utilities for these two studies ranged from 0.56 to 0.89. Two studies^32^, ^34^ analysed cancers of specific anatomical locations using EQ‐5D with utility values of 0.56–0.65 (treated) for the lower limb and 0.72 (untreated) for the head and neck. One study^43^ focussed on cancers of the face and ears and obtained utilities using the time trade‐off method of 0.97–1 (treated). Utilities for patients with lesions of suspected but unconfirmed malignant potential ranged from 0.97 to 0.98.^41^

**TABLE 2:** Health utilities for undifferentiated non‐melanoma skin cancer

Authors	Health States	Subgroup Numbers	Mean Utility Value (Median)	Standard Deviation	Method
**Grouped by NMSC, untreated or where treatment is unspecified**					
Chen et al	NMSC	8	0.976 (1.000)	0.052	TTO
Littenberg et al	NMSC	8	0.995		SG
Philipp‐Dormston et al	BCC and SCC in the same participant (including those also with AK)	82	0.80 (0.89)		EQ 5D 5L^b^
Wali et al	NMSC	259	0.88	0.18	EQ 5D 5L
Bertino et al	Head and neck cancer (baseline)	105	0.72 (0.80)		EQ 5D
Sullivan, Ghushchyan	Other malignant neoplasm of the skin (mean age = 67)	453	0.812 (0.816)		EQ 5D 3L
Sullivan, Ghushchyan	Benign neoplasm of the skin (mean age = 49)	443	0.861 (0.827)		EQ 5D 3L
Sullivan, Slejko et al	Other non‐epithelial cancer of the skin (mean age = 66)	1026	0.765 (0.796)	0.009^a^	EQ 5D 3L
Sullivan, Slejko et al	Other malignant neoplasm of the skin (mean age = 66.2)	918	0.757 (0.796)	0.010^a^	EQ 5D 3L
Sullivan, Slejko et al	Benign neoplasm of the skin (mean age = 49.1)	902	0.827 (0.814)	0.008^a^	EQ 5D 3L
**Grouped by treated NMSC, where treatment is specified**					
Ker et al	Standard dressing, 5–7 days post‐treatment	19	0.646	0.263	EQ 5D 3L
Ker et al	Negative‐pressure wound therapy, 5–7 days post‐treatment	28	0.563	0.184	EQ 5D 3L
Seidler, Bramlette et al	Mohs surgery or traditional surgical excision	98	0.996		TTO
Seidler, Bramlette et al	Simple closure (granulation or primary closure)	98	0.984		TTO
Seidler, Bramlette et al	Complex closure (flap or graft)	98	0.974		TTO
Seidler, Bramlette et al	Recurrence of cancer	98	0.984		TTO
Bertino et al	1 month post‐treatment (electrochemotherapy and bleomycin)		0.71 (0.80)		EQ 5D
Bertino et al	2 months post‐treatment (electrochemotherapy and bleomycin)	91	0.74 (0.80)		EQ 5D
Bertino et al	4 months post‐treatment (electrochemotherapy and bleomycin)	72	0.79 (0.85)		EQ 5D
Bertino et al	8 months post‐treatment (electrochemotherapy and bleomycin)	52	0.85 (0.85)		EQ 5D
Bertino et al	12 months post‐treatment (electrochemotherapy and bleomycin)	36	0.89 (0.94)		EQ 5D
**Miscellaneous**					
Chen et al	Rule out NMSC	10	0.979 (0.997)	0.036	TTO
Chen et al	Neoplasia of uncertain behaviour (lesion biopsied and awaiting result)	35	0.971 (0.996)	0.047	TTO
Chen et al	Benign tumour	17	0.974 (1.000)	0.054	TTO
Chen et al	Rule out malignant melanoma and dysplastic nevi	11	0.979 (0.988)	0.026	TTO

Abbreviations: NMSC, non‐melanoma skin cancer; BCC, basal cell carcinoma; SCC, squamous cell carcinoma; AK, actinic keratosis; EQ 5D, EuroQol 5‐dimension instrument; TTO, time trade‐off; SG, standard gamble.

^a^ Standard error.

^b^ The EQ 5D 5L is the 5‐level version of the EQ 5D 3L, the 3‐level questionnaire.^70^

#### 4.3.2 Actinic keratosis

Six studies provided health states for AK (Table 3).^33^, ^35^, ^39^, ^41^, ^42^, ^46^ Mean utility ranges were 0.84 to 0.99 and 0.89 to 0.96 for untreated AK (or where treatment unspecified) and treated AK, respectively. The utility for patients with pruritis was 0.92 (SD = 0.15),^41^ which was included due to pruritis being a common side effect of topical treatments for AK.

**TABLE 3:** Health utilities for actinic keratosis

Authors	Health States	Subgroup Numbers	Mean Utility Value (Median)	Standard Deviation	Method
**Grouped by AK, untreated or where treatment is unspecified**					
Chen et al	AK	9	0.981 (1.000)	0.056	TTO
Hanke et al	AK, baseline for overall treatment group	329	0.927		EQ 5D 3L
Hanke et al	AK, baseline for cryotherapy treatment group	162	0.92^a^ (1.00)	0.11	EQ 5D 3L
Hanke et al	AK, baseline for cryotherapy and ingenol mebutate treatment group	167	0.93^a^ (1.00)	0.10	EQ 5D 3L
Littenberg et al	AK	16	0.989		SG
Philipp‐Dormston et al	AK (single diagnosis)	468	0.89 (1.00)		EQ 5D 5L
Seidler, Bayoumi et al	AK	7	(1.00)		TTO
Tennval et al	Current AK	244	0.881		EQ 5D 5L
Tennval et al	Current AK (face)	170	0.884		EQ 5D 5L
Tennval et al	Current AK (non‐facial)	74	0.873		EQ 5D 5L
Tennval et al	Current AK (immunosuppressive treatment for other conditions)	23	0.876		EQ 5D 5L
Tennval et al	Severe actinic damage	26	0.844		EQ 5D 5L
Tennval et al	Current AK (with suspected NMSC)	37	0.856		EQ 5D 5L
Tennval et al	Current AK (with previous SCC)	51	0.849		EQ 5D 5L
**Grouped by treated AK, where treatment is specified**					
Hanke et al	AK, 8 weeks post‐treatment for overall treatment group	304	0.960		EQ 5D 3L
Hanke et al	Cryotherapy – 1 day post‐treatment	162	0.91^a^ (1.00)	0.08	EQ 5D 3L
Hanke et al	Cryotherapy – 3 weeks post‐treatment	153	0.94^a^ (1.00)	0.09	EQ 5D 3L
Hanke et al	Cryotherapy – 3 weeks and 3 days post‐treatment	148	0.96^a^ (1.00)	0.09	EQ 5D 3L
Hanke et al	Cryotherapy – 5 weeks post‐treatment	147	0.96^a^ (1.00)	0.10	EQ 5D 3L
Hanke et al	Cryotherapy – 11 weeks post‐treatment	148	0.95^a^ (1.00)	0.13	EQ 5D 3L
Hanke et al	Cryotherapy – 6 months post‐treatment	148	0.95^a^ (1.00)	0.11	EQ 5D 3L
Hanke et al	Cryotherapy – 12 months post‐treatment	140	0.95^a^ (1.00)	0.10	EQ 5D 3L
Hanke et al	Cryotherapy and ingenol mebutate – 1 day post‐treatment	166	0.92^a^ (1.00)	0.08	EQ 5D 3L
Hanke et al	Cryotherapy and ingenol mebutate – 3 weeks post‐treatment	161	0.95^a^ (1.00)	0.09	EQ 5D 3L
Hanke et al	Cryotherapy and ingenol mebutate – 3 weeks and 3 days post‐treatment	156	0.89^a^ (1.00)	0.12	EQ 5D 3L
Hanke et al	Cryotherapy and ingenol mebutate – 5 weeks post‐treatment	155	0.96^a^ (1.00)	0.11	EQ 5D 3L
Hanke et al	Cryotherapy and ingenol mebutate – 11 weeks post‐treatment	156	0.96^a^ (1.00)	0.10	EQ 5D 3L
Hanke et al	Cryotherapy and ingenol mebutate – 6 months post‐treatment	153	0.96^a^ (1.00)	0.10	EQ 5D 3L
Hanke et al	Cryotherapy and ingenol mebutate – 12 months post‐treatment	149	0.95^a^ (1.00)	0.09	EQ 5D 3L
Tennval et al	Current AK treatment	120	0.900		EQ 5D 5L
Chen et al	Pruritis and related conditions	5	0.915 (0.966)	0.145	TTO

Abbreviations: NMSC, non‐melanoma skin cancer; SCC, squamous cell carcinoma; AK, actinic keratosis; EQ 5D, EuroQol 5‐dimension instrument; TTO, time trade‐off; SG, standard gamble.

^a^ Utility values obtained from unpublished data.

#### 4.3.3 Squamous cell carcinoma

Health states pertaining to SCC were examined in four studies (Table 4).^35^, ^36^, ^45^, ^47^ Mean utilities ranged from 0.63 to 0.99, 0.97 to 0.99, and 0.45 to 1 for people with untreated SCC (or where treatment unspecified), SCC treated with radiotherapy, and SCC treated by other treatments, respectively.

**TABLE 4:** Health utilities for squamous cell carcinoma

Authors	Health States	Subgroup Numbers	Mean Utility Value (Median)	Standard Deviation	Method
**Grouped by SCC, untreated or where treatment is unspecified**					
Lear et al	SCC	41	0.99	0.003	SG
Philipp‐Dormston et al	SCC (single diagnosis, but including participants with both SCC and AK)	112	0.84 (0.91)		EQ 5D 5L
Philipp‐Dormston et al	SCC (single diagnosis)	32	0.90 (0.91)		EQ 5D 5L
Philipp‐Dormston et al	SCC and AK (in the same participant)	80	0.82 (0.91)		EQ 5D 5L
Pil et al	SCC, stage 0–II (undiagnosed)		0.812		–‐^a^
Pil et al	SCC, stage III (undiagnosed)		0.631		–‐^b^
Pil et al	SCC, stage IV (undiagnosed)		0.651		–‐^b^
**Grouped by treated SCC (radiotherapy)**					
Wong et al	Nodal dissection and radiotherapy	14	0.9700	0.0400	SG
Wong et al	Radiotherapy alone	7	0.980	0.010	SG
Lear et al	SCC + radiation	41	0.99	0.01	SG
**Grouped by treated SCC (other treatments)**					
Wong et al	Salvage of recurrence following initial elective treatment	2	0.94	0.05	SG
Wong et al	Nodal dissection alone	7	0.99	0.01	SG
Lear et al	SCC + electrodesiccation and curettage	41	0.98	0.08	SG
Lear et al	SCC + excision	41	0.999	0.002	SG
Lear et al	SCC + Mohs surgery	41	1.0000	0.0002	SG
Pil et al	SCC, stage 0–II (diagnosis and treatment)	7	0.532		EQ 5D
Pil et al	SCC, stage 0–II (intense follow‐up)	11	0.707		EQ 5D
Pil et al	SCC, stage 0–II (long‐term follow‐up)		0.812		–‐^a^
Pil et al	SCC, stage III (diagnosis and treatment)		0.450		–‐^c^
Pil et al	SCC, stage III (intense follow‐up)		0.620		–‐^c^
Pil et al	SCC, stage III (long‐term follow‐up)		0.706		–‐^c^
Pil et al	SCC, stage IV (diagnosis and treatment)		0.490		–‐^c^
Pil et al	SCC, stage IV (intense follow‐up)		0.702		–‐^c^
Pil et al	SCC, stage IV (long‐term follow‐up)		0.799		–‐^c^

Abbreviations: SCC, squamous cell carcinoma; AK, actinic keratosis; EQ 5D, EuroQol 5‐dimension instrument; SG, standard gamble.

^a^ These subgroups were assigned the same utility as the Belgium population norm.

^b^ Utilities calculated for these subgroups as the average of the population norm and the utility for diagnosis and treatment.

^c^ Utilities calculated for these subgroups based on the ratio of utilities in these stages compared to stage I – authors referred to Tromme et al^71^ for calculation method.

Pil et al^36^ obtained utilities for SCC stratified by stage at diagnosis. Stage 0–II, stage III, and stage IV produced utility values of 0.53, 0.45, and 0.49, respectively, and intense follow‐up of treated SCC stage 0–II, stage III, and stage IV of 0.71, 0.62, and 0.70, respectively. The type of treatment was not specified in this study.

#### 4.3.4 Basal cell carcinoma

Six studies examined health states for BCC (Table 5).^35^, ^36^, ^41^, ^42^, ^44^, ^45^ Ranges of mean utilities were 0.67 to 1, 0.72 to 1, and 0.82 to 1 for untreated BCC (or where treatment was unspecified), BCC treated with physical treatments, and BCC treated with other treatments, respectively. The utility for suspected but unconfirmed BCC was 0.97 (SD = 0.04).^41^ For advanced BCC, Shingler et al^44^ found utility values ranged from 0.67 (SD = 0.25) for progressed disease with 6 cm growth to 0.94 (SD = 0.08) after complete treatment response.

**TABLE 5:** Health utilities for basal cell carcinoma

Authors	Health States	Subgroup Numbers	Mean Utility Value (Median)	Standard Deviation	Method
**Grouped by BCC, untreated or where treatment is unspecified**					
Lear et al	BCC	41	0.999	0.003	SG
Pil et al	BCC undiagnosed		0.812		–‐^a^
Pil et al	BCC (diagnosed)		0.790		–‐^b^
Pil et al	BCC (intense follow‐up)		0.790		–‐^b^
Pil et al	BCC (long‐term follow‐up)		0.812		–‐^a^
Philip‐Dormston et al	BCC (single diagnosis, but including participants with both BCC and AK)	472	0.87 (0.91)		EQ 5D 5L
Seidler, Bayoumi et al	BCC	5	– (0.95)		TTO
Shingler et al	Advanced BCC (stable disease with small growth – 2 cm)	100	0.82	0.16	TTO
Shingler et al	Advanced BCC (stable disease with multiple growths – 2 cm)	100	0.80	0.20	TTO
Shingler et al	Advanced BCC (stable disease with large growth – 6 cm)	100	0.76	0.20	TTO
Shingler et al	Advanced BCC (progressed disease with small growth – 2 cm)	100	0.74	0.21	TTO
Shingler et al	Advanced BCC (progressed disease with large growth – 6 cm)	100	0.67	0.25	TTO
**Grouped by treated BCC (physical treatments)**					
Lear et al	BCC + electrodesiccation and curettage	41	0.999	0.003	SG
Lear et al	BCC + excision	41	0.999	0.002	SG
Lear et al	BCC + Mohs surgery	41	1.00000	0.0001	SG
Shingler et al	Advanced BCC (post‐surgical state)	100	0.72	0.24	TTO
**Grouped by treated BCC (other treatments)**					
Lear et al	BCC + radiation	41	0.999	0.003	SG
Shingler et al	Advanced BCC (complete treatment response)	100	0.94	0.08	TTO
Shingler et al	Advanced BCC (partial response with small growth – 2 cm)	100	0.88	0.12	TTO
Shingler et al	Advanced BCC (partial response with large growth – 6 cm)	100	0.82	0.16	TTO
**Miscellaneous**					
Chen et al	Rule out BCC	8	0.974 (0.997)	0.04	TTO

Abbreviations: BCC, basal cell carcinoma; AK, actinic keratosis; EQ 5D, EuroQol 5‐dimension instrument; TTO, time trade‐off; SG, standard gamble.

^a^ These subgroups were assigned the same utility as the Belgium population norm.

^b^ Derived from Gaulin et al.^72^

#### 4.3.5 Utilities pre‐ and post‐treatment

Five studies compared pre‐ and post‐treatment health states, with generally higher utility values post‐treatment.^32^, ^33^, ^36^, ^39^, ^45^ Treatment subgroups, where specified, included surgical excision (simple excision, flap excision, and Mohs surgery), cryotherapy, ingenol mebutate, electrochemotherapy and bleomycin, radiation therapy, and nodal dissection. Serial measurements in three studies^32^, ^33^, ^36^ showed an initial decrease in utility following initiation of treatment, which increased over the post‐treatment period and reached or exceeded the baseline utility value. Two of these studies^32^, ^33^ measured repeated utility values at defined time periods after treatment.

Overall, untreated stage III and stage IV SCC and untreated advanced BCC produced the lowest utility values, whereas utilities obtained in patients with AK were significantly higher.

### 4.4 Valuation methods

#### 4.4.1 EuroQol 5‐dimension instrument

Nine studies used the EQ‐5D questionnaire to measure health utility, which ranged from means of 0.45 to 0.96.^32^, ^33^, ^34^, ^35^, ^36^, ^37^, ^38^, ^39^, ^40^ Five of these studies were conducted among patients with undifferentiated NMSC without further subgroup analysis.^32^, ^34^, ^37^, ^38^, ^40^ One study reported on undifferentiated NMSC as well as separate subgroup analysis of AK, BCC, and SCC.^35^ Two studies reported on AK,^33^, ^39^ and one focussed on SCC.^36^ Utilities obtained for pre‐treatment AK subgroups ranged from 0.88 to 0.93.^33^, ^35^, ^39^ Utilities for undifferentiated NMSC (untreated or treatment unspecified) ranged from 0.72 to 0.88.^32^, ^35^, ^37^, ^38^, ^40^

#### 4.4.2 Standard gamble

Three studies^45^, ^46^, ^47^ used the standard gamble method, which produced consistently high mean utility values ranging from 0.94 to 1. One study^45^ in a skin cancer clinic used hypothetical scenarios rather than the participant’s current health state. Two studies^45^, ^47^ reported utilities for SCC patients following radiation therapy: One study reported utility of 0.99 (SD = 0.003) for a hypothetical primary SCC of the lip and the other reported utility of 0.97 to 0.98 for patients with advanced or metastatic SCC of the head and neck.

#### 4.4.3 Time trade‐off

Four studies^41^, ^42^, ^43^, ^44^ obtained mean utility values using the time trade‐off method with values ranging from 0.67 to 1. One study^44^ used standardized clinical vignettes presented to the healthy general population with utilities ranging from 0.67 to 0.94. Three studies^41^, ^42^, ^43^ applied the method to the patient’s actual condition with mean utilities ranging from 0.92 to 1.

## 5 DISCUSSION

There is considerable variability in the reported health utilities for NMSCs, depending on type of cancer, stage of diagnosis, time since treatment administered, type of treatment, other characteristics of the cancer, and method of utility valuation. Despite methodological differences between studies, there is broad consistency of findings with a priori expectations for a condition that usually does not cause severe symptoms or death. Mean utility values ranged from 0.45 to 1 but most were clustered around relatively high values of 0.8 to 1 reflecting less disease burden or the use of methods that lead to smaller estimates of disutility. Some of the lowest utility values (range 0.45 to 0.65) corresponded with advanced stages of SCC and patients requiring split‐skin grafting for lower limb cancers, a treatment normally used for large cancers. Low utility values were also found for treatment of advanced BCC. It is estimated that metastatic and locally advanced NMSC account for approximately 0.3% and 0.9% of the total annual incidence rate of NMSC, respectively.^48^ Complex surgical repairs are common^49^ and are used for tumours with high‐risk features or on complex anatomical sites, with the inherent increased risk of complications.^50^, ^51^ Despite AK being a precursor (non‐malignant) lesion, there are some health states for AK that have similar utilities to that of NMSC, which suggests that the measures may not be sufficiently specific to distinguish between the various skin tumour types. There was marked heterogeneity of the different treatments used across the studies.

Mean utility values of single‐cancer subgroups across the studies were 0.56 to 1 for NMSC, 0.84 to 0.99 for AK, 0.45 to 1 for SCC, and 0.67 to 1 for BCC, respectively. For comparison, published mean pooled utility weights for stage I/II, stage III, and stage IV melanoma are 0.97, 0.77, and 0.76, respectively^52^ and for psoriasis, 0.91.^41^ We did not meta‐analyse the primary data since there was considerable heterogeneity regarding stage diagnosed, time since treatment, treatment type, and utility instrument or method. It is likely that anatomic location of the cancer influences the quality of life,^53^, ^54^ however, only a few studies reported this. The context of each study, such as the population demographics^55^ and anatomic location, is an important consideration when selecting which utility estimates to use, so caution should be used when pooling utility values for economic analyses.^56^

The method or instrument also influenced the utility weights reported. Higher values were found in studies using the standard gamble method, which could be attributed to the endowment effect^57^ – a tendency for higher utility values due to the healthy general population’s aversion to succumbing to illness, limited understanding of the natural history, or the influence of the presented clinical scenarios to the outcomes.^45^ A similar effect was also noted using the time trade‐off method.^44^ The asymmetry of information and difference in the understanding of the disease process between health professionals and patients highlights the importance of piloting the feasibility of the valuation approaches, and the challenges with establishing a single value to a temporary and changing health state.^58^

Only one study^35^ measured the marginal utility of increasing cancer burden on patients, which compared the mean utility between participants with either BCC (0.87) or SCC (0.84) against participants with both BCC and SCC (0.80), a difference of 0.07 and 0.04, respectively. Similarly the mean utility for participants with a single diagnosis of AK (0.89) and SCC (0.90) was compared to participants with both AK and SCC (0.82), a difference of 0.07 and 0.08, respectively.^35^ Since many patients develop multiple tumours and require ongoing monitoring and treatment, the experience of skin cancer is akin to a chronic disease and is not a temporary minor ailment as commonly portrayed.^35^, ^59^

Participants were drawn from countries with predominantly fair‐skin populations, however there were no studies from Australia, which has the highest rates of skin cancer in the world.^1^ The New Zealand studies^34^, ^47^ had small sample sizes and only studied health utilities for specific treatment modalities related to treatment of large skin cancers. Some of the excluded studies^60^, ^61^ used pooled utilities derived from other studies and applied the value to populations and countries distinct from the original sample population. Generalizability of utility values between different populations can be adequately assessed when there is demographic data and clearly defined health states based on clinical characteristics.

Six studies^34^, ^43^, ^44^, ^45^, ^46^, ^47^ had a sample size of under 100. In particular, Pil et al^36^ used assigned baseline utility values and extrapolation of utilities for SCC stages II, III, and IV (diagnosed) due to insufficient sample size. The two national population studies^37^, ^38^ had large subgroups of over 400 participants with NMSC, however the pooling of data limits further disease‐specific analysis. Philipp‐Dormston et al’s study^35^ recruited 1184 participants from hospitals and local medical practices in Germany and was assessed as low risk of bias, therefore, based on these factors, could be considered to have produced reliable utility values.

### 5.1 Limitations

Studies that reported mapped utilities or used instruments mapped by an algorithm to EQ‐5D were excluded. For health economic modelling purposes, it is preferable to use utility values obtained directly from instruments and to reserve mapped values for when such data is not available.^62^ Intrinsically the quality of mapped values relies on the accuracy of mapping algorithms. Also, non‐utility based measures such as the DLQI may be more suitable for chronic, benign skin conditions such as eczema than NMSC as the instrument is sensitive to changes in level of discomfort related to itchiness and irritation, but not to treatment‐related scarring or disfigurement nor to patient anxiety about recurrence.^63^, ^64^, ^65^, ^66^, ^67^, ^68^ However, instruments that have not yet been mapped to utility values, such as Skindex‐16,^17^ the Skin Cancer Index,^69^ and BaSQoL, which are NMSC‐specific and include measures of sun protective behaviours after skin cancer diagnosis and worries about treatment,^18^ may also yield further insights into quality of life when compared to generic tools, such as EQ‐5D, where subtleties of the quality of life experience may be lost.^35^ One of the key advantages of generic tools is the transferability of results to health economics studies. Hence, there may be value in conducting further research in maximizing the sensitivity of quality of life data that are used in health economics studies.

Studies in languages other than English were excluded and although this would represent few studies, it is possible that NMSC utilities differ by race. Authors of 12 short‐listed conference abstracts were unable to provide further information, so only limited information could be extracted for these.

Overall, this review highlights the paucity of evidence in the literature, with only seven studies^35^, ^36^, ^41^, ^42^, ^44^, ^45^, ^47^ that reported utility values specifically for patients with BCC or SCC, suggesting further research is needed to obtain accurate and reliable utility values. Future primary research should aim for larger sample sizes with a priori specification of the required sample sizes for assessing utilities to a prespecified level of precision and report utilities for different subgroups defined by age, anatomic location, treatment status and the period of time post‐treatment due to the impact of disfigurement and discomfort.^53^ Patient comorbidity may also have an impact on the quality of life, so baseline clinical information would be useful information. Early detection of skin tumours leads to improved outcomes in most cases so future research should aim to demonstrate the likely higher post‐treatment utility values where skin tumours have been treated early.

In conclusion, this systematic review found that most health utilities clustered around relatively high mean values of 0.8 to 1 for NMSCs and their precursors, with lower utility values for more advanced cancers. Although there are considerable difficulties comparing values obtained from studies using different methods, this seems to indicate small decrements to quality of life associated with the clinical management of most NMSC and AK. There was substantial variability in the utilities for different skin cancer subtypes, treatments and methods. Developing an accurate and comprehensive catalogue of NMSC health utilities in different populations is crucial for future health economic evaluations to adequately inform policy and resource allocation decisions related to skin cancer early detection and management.

## CONFLICTS OF INTEREST

The authors have no conflicts of interest to declare.

## Supporting information

Suppoting Information S1
