# Supplementary material for: Health utilities for non‐melanoma skin cancers and pre‐cancerous lesions: A systematic review
Source: Skin Health Dis. 2021 Jun 4;1(3):e51. doi: 10.1002/ski2.51 (PMC9060093; doi:10.1002/ski2.51)
Supplement: Supplementary file 1 — Suppoting Information S1 [file SKI2-1-e51-s001.docx]

**Supporting Information**

| **Table S1. Search strategy for Medline.** | |
| --- | --- |
| 1  2  3  4  5  6  7  8  9  10  11  12  13  14  15  16  17  18  19  20  21  22  23  24 | keratinocyte cancer.tw.  neoplasms, basal cell/ or carcinoma, basal cell/  neoplasms, squamous cell/ or carcinoma, squamous cell/ or bowen's disease/  non melanoma skin cancer.tw.  nonmelanoma skin cancer.tw.  basal cell.tw.  Keratosis, Actinic/  actinic keratos*.tw.  solar keratos*.tw.  ("squamous cell" adj5 (skin or cutaneous)).tw.  1 or 2 or 3 or 4 or 5 or 6 or 7 or 8 or 9  (1 or 2 or 3 or 4 or 5 or 6 or 7 or 8 or 9) and (skin or cutaneous).tw.  exp quality of life/  (Euroqol or eq5d or eq 5d).tw.  standard gamble.tw.  health utilit$.tw.  hui.tw.  (time trade off or time trade-off).tw.  Quality adjusted life year$.tw.  Qaly$.tw.  13 or 14 or 15 or 16 or 17 or 18 or 19 or 20  10 or 12  21 and 22  limit 23 to (human and english language) |

**Appendix S1**

**Cochrane Database of Systematic Reviews search strategy**

Title Abstract Keyword “non melanoma skin” OR “keratinocyte”

AND Title Abstract Keyword “quality of life”

**Figure S1. Results from search strategy**

**Initial search results:**

Medline 145

Embase 440

Cochrane 4

Title and abstract reviewed

589

**Title and abstract review**

**Exclusions:**

Duplicates 113

Not relevant to NMSC 137

No QOL instrument 141

Non-utility based QOL instrument 82

Potentially relevant studies

116

Conference abstracts 23

**Authors contacted for further information and data:**

Duplicate (full text already produced in search) 5

Response – health utility available 0

Response – no health utility 6

No response 9

Unable to contact 3

Full text reviewed

93

**Full text review**

**Exclusions:**

Not relevant to NMSC 7

No QOL instrument 23

Non-utility based QOL instrument 38

Utility from secondary source 14

Studies that met criteria 16

5 additional relevant studies identified through hand-searching of reference lists of relevant studies

| **Table S2. Risk of bias assessment based on ROBINS-I checklist for missing data.** | | | | | | | |
| --- | --- | --- | --- | --- | --- | --- | --- |
| **First author studies** | **Questions 5.1**  **Were outcome data available for all, or nearly all, participants?**  **Y/PY / PN / N / NI** | **Question 5.2**  **Were participants excluded due to missing data on intervention status?**  **Y/PY / PN / N / NI** | **Question 5.3**  **Were participants excluded due to missing data on other variables needed for the analysis?**  **Y/PY / PN / N / NI** | **Question 5.4**  **If PN/N to 5.1, or Y/PY to 5.2 or 5.3: Are the proportion of participants and reasons for missing data similar across interventions?**  **Y/PY / PN / N / NI** | **Question 5.5**  **If PN/N to 5.1, or Y/PY to 5.2 or 5.3: Is there evidence that results were robust to the presence of missing data?**  **Y/PY / PN / N / NI** | **Optional**  **What is the predicted direction of bias due to missing data?** | **Missing data risk of bias judgement**  **Low / Moderate / Serious / Critical / NI** |
| Bertino et al | Y (94%) | N | PN (6/105 excluded as required follow up ‘was not possible’) | NA | NA | Unpredictable | Low |
| Chen et al | PY (88-96% across different sites) | No intervention | Y (noncompleters and participants who did not understand TTO or gave inconsistent responses were excluded) | No intervention | PY (high completion rate across sites) | Unpredictable | Low |
| Hanke et al | Y (92% of total participants) | N | Y (26/329 missing) | Y (baseline EQ5D values among excluded participants did not differ significantly) | Y | Unpredictable | Low |
| Ker et al | Y (96% of total participants) | N | Y (2/49 missing) | PN (very low numbers of missing data) | PY (due to low numbers of missing data) | Unpredictable | Low |
| Lear et al | NI (patients excluded if unable to consent but no indication of how many excluded) | No intervention | N | NA | NA | Unpredictable | NI |
| Littenberg et al | N (36%) | No intervention | Y (2 uninterpretable results, 6 observed misordering, 3 answered no to all questions) | No intervention | N | Unpredictable | Serious |
| Philipp-Dormston et al | Y (10/1194 excluded) | No intervention | Y (22/1184 noncompletion) | No intervention | PY (high completion rate) | Unpredictable | Low |
| Pil et al | N (response rates 83% in dermatology and 72% oncology patients) | N | NI | NI | NI | Unpredictable | Serious |
| Seidler, Bayoumi et al | N (response rates ranged from 25% to 97% depending on site) | No intervention | Y (29/283 excluded due to inconsistent or uninterpretable responses) | No intervention | NI (although only 10% of recruited participants were excluded due to suboptimal responses) | Unpredictable | Moderate |
| Seidler, Bramlette et al | Y (100%) | N | N | NA | NA | NA | Low |
| Shingler et al | PY (100/100 however no information regarding those who declined consent; sample matched UK general public demographics) | No intervention | N | NA | NA | Unpredictable | Low |
| Sullivan, Ghuschyan | Y (nationally representative survey) | No intervention | NI | No intervention | NA | Unpredicatable | Low |
| Sullivan, Slejko et al | Y (nationally representative survey) | No intervention | NI | No intervention | NA | Unpredicatable | Low |
| Tennval et al | PY (88%) | No intervention | Y (36/312) | No intervention | PY (high response rate) | Unpredictable | Low |
| Wali et al | N (259/318, 81%) | No intervention | Y | No intervention | NI | Unpredictable | Moderate |
| Wong et al | Y (30/30, 100%) | N | N | NA | NA | NA | Low |

Y, yes; PY, probably yes; PN, probably no; N, no; NI, no information; NA, not applicable.

**Table S3. PRISMA Checklist**

| **Section/topic** | **#** | **Checklist item** | **Reported on page #** |
| --- | --- | --- | --- |
| **TITLE** | | |  |
| Title | 1 | Identify the report as a systematic review, meta-analysis, or both. | 1 |
| **ABSTRACT** | | |  |
| Structured summary | 2 | Provide a structured summary including, as applicable: background; objectives; data sources; study eligibility criteria, participants, and interventions; study appraisal and synthesis methods; results; limitations; conclusions and implications of key findings; systematic review registration number. | 3 |
| **INTRODUCTION** | | |  |
| Rationale | 3 | Describe the rationale for the review in the context of what is already known. | 5 |
| Objectives | 4 | Provide an explicit statement of questions being addressed with reference to participants, interventions, comparisons, outcomes, and study design (PICOS). | 6 |
| **METHODS** | | |  |
| Protocol and registration | 5 | Indicate if a review protocol exists, if and where it can be accessed (e.g., Web address), and, if available, provide registration information including registration number. | 7 |
| Eligibility criteria | 6 | Specify study characteristics (e.g., PICOS, length of follow-up) and report characteristics (e.g., years considered, language, publication status) used as criteria for eligibility, giving rationale. | 7 |
| Information sources | 7 | Describe all information sources (e.g., databases with dates of coverage, contact with study authors to identify additional studies) in the search and date last searched. | 6-7 |
| Search | 8 | Present full electronic search strategy for at least one database, including any limits used, such that it could be repeated. | Supp file |
| Study selection | 9 | State the process for selecting studies (i.e., screening, eligibility, included in systematic review, and, if applicable, included in the meta-analysis). | 7 |
| Data collection process | 10 | Describe method of data extraction from reports (e.g., piloted forms, independently, in duplicate) and any processes for obtaining and confirming data from investigators. | 7 |
| Data items | 11 | List and define all variables for which data were sought (e.g., PICOS, funding sources) and any assumptions and simplifications made. | 7 |
| Risk of bias in individual studies | 12 | Describe methods used for assessing risk of bias of individual studies (including specification of whether this was done at the study or outcome level), and how this information is to be used in any data synthesis. | 7 |
| Summary measures | 13 | State the principal summary measures (e.g., risk ratio, difference in means). | 7 |
| Synthesis of results | 14 | Describe the methods of handling data and combining results of studies, if done, including measures of consistency (e.g., I^2^) for each meta-analysis. | 7 |

| **Section/topic** | **#** | **Checklist item** | **Reported on page #** |
| --- | --- | --- | --- |
| Risk of bias across studies | 15 | Specify any assessment of risk of bias that may affect the cumulative evidence (e.g., publication bias, selective reporting within studies). | 14-15 |
| Additional analyses | 16 | Describe methods of additional analyses (e.g., sensitivity or subgroup analyses, meta-regression), if done, indicating which were pre-specified. | 7 |
| **RESULTS** | | |  |
| Study selection | 17 | Give numbers of studies screened, assessed for eligibility, and included in the review, with reasons for exclusions at each stage, ideally with a flow diagram. | 7-8, Supp file |
| Study characteristics | 18 | For each study, present characteristics for which data were extracted (e.g., study size, PICOS, follow-up period) and provide the citations. | 8 |
| Risk of bias within studies | 19 | Present data on risk of bias of each study and, if available, any outcome level assessment (see item 12). | 9 |
| Results of individual studies | 20 | For all outcomes considered (benefits or harms), present, for each study: (a) simple summary data for each intervention group (b) effect estimates and confidence intervals, ideally with a forest plot. | 9-12 |
| Synthesis of results | 21 | Present results of each meta-analysis done, including confidence intervals and measures of consistency. | NA |
| Risk of bias across studies | 22 | Present results of any assessment of risk of bias across studies (see Item 15). | 14-15 |
| Additional analysis | 23 | Give results of additional analyses, if done (e.g., sensitivity or subgroup analyses, meta-regression [see Item 16]). | 9-12 |
| **DISCUSSION** | | |  |
| Summary of evidence | 24 | Summarize the main findings including the strength of evidence for each main outcome; consider their relevance to key groups (e.g., healthcare providers, users, and policy makers). | 13 |
| Limitations | 25 | Discuss limitations at study and outcome level (e.g., risk of bias), and at review-level (e.g., incomplete retrieval of identified research, reporting bias). | 14-15 |
| Conclusions | 26 | Provide a general interpretation of the results in the context of other evidence, and implications for future research. | 15 |
| **FUNDING** | | |  |
| Funding | 27 | Describe sources of funding for the systematic review and other support (e.g., supply of data); role of funders for the systematic review. | 2 |

*From:*  Moher D, Liberati A, Tetzlaff J, Altman DG, The PRISMA Group (2009). Preferred Reporting Items for Systematic Reviews and Meta-Analyses: The PRISMA Statement. PLoS Med 6(7): e1000097. doi:10.1371/journal.pmed1000097

For more information, visit: **www.prisma-statement.org**.
